# Early Visual Processing of Feature Saliency Tasks: A Review of Psychophysical Experiments

**DOI:** 10.3389/fnsys.2018.00054

**Published:** 2018-10-26

**Authors:** Shiva Kamkar, Hamid Abrishami Moghaddam, Reza Lashgari

**Affiliations:** ^1^Machine Vision and Medical Image Processing Laboratory, Faculty of Electrical and Computer Engineering, K. N. Toosi University of Technology, Tehran, Iran; ^2^Brain Engineering Research Center, Institute for Research in Fundamental Sciences (IPM), Tehran, Iran

**Keywords:** psychophysics, feature saliency, visual task, visual attention, primary visual cortex

## Abstract

The visual system is constantly bombarded with information originating from the outside world, but it is unable to process all the received information at any given time. In fact, the most salient parts of the visual scene are chosen to be processed involuntarily and immediately after the first glance along with endogenous signals in the brain. Vision scientists have shown that the early visual system, from retina to lateral geniculate nucleus (LGN) and then primary visual cortex, selectively processes the low-level features of the visual scene. Everything we perceive from the visual scene is based on these feature properties and their subsequent combination in higher visual areas. Different experiments have been designed to investigate the impact of these features on saliency and understand the relative visual mechanisms. In this paper, we review the psychophysical experiments which have been published in the last decades to indicate how the low-level salient features are processed in the early visual cortex and extract the most important and basic information of the visual scene. Important and open questions are discussed in this review as well and one might pursue these questions to investigate the impact of higher level features on saliency in complex scenes or natural images.

## Introduction

The visual cortex is constantly processing the information coming from the outside world as well as endogenous signals originating from higher brain levels. The information coming from the outside world contains cluttered visual scenes that must be parsed to identify meaningful objects. However, the visual cortex has limited capacity to process all this information at any given time. This limitation is mostly related to the timing and physiological properties of neurons, visual spatial mapping, and their neuronal circuits that are tuned to specific features of visual scenes (Shiffrin and Gardner, 1972; Tsotsos, 1997; Kastner and Ungerleider, 2000). It can also be influenced by intrinsic brain states. A principal approach that the brain uses to defy this capacity limitation is to attend involuntarily to the salient parts at the first glance. In addition, it can voluntarily select specific parts of the visual scene by making use of intrinsic signals such as expectations, attention, memories, and behavioral states (Motter, 1993; Desimone and Duncan, 1995; Maunsell, 1995; Pashler, 1998; Peck et al., 2009). Therefore, the perceived visual scenes are scanned more efficiently by combining neuronal filters with cognitive signals that guide the scanning process. Attention is a fundamental mechanism to control the processing of visual information. Selective visual attention is either the process of focusing on a part of the visual scene in the center of gaze, within the high acuity fovea which has a high density of photoreceptors, or covertly attending at the locations away from the center of gaze, where visual resolution is low. This mechanism is important since the processing of the entire visual scene is beyond the ability of the brain. Consequently, selective attention allows us to tune out the unimportant visual information and focus on what information really matters which is embedded in the salient part(s) of the scene (Wolf and Horowitz, 2004; Carrasco, 2011).

Saliency is mainly a stimulus driven process (Nordfang and Wolfe, 2014). The salient part of the scene stands out from its surround because of a difference in one or more physical factors (Treue, 2003; Töllner et al., 2011; Krüger et al., 2016) due to discontinuities (Borji et al., 2014) or lack of correlation (Blake and Lee, 2005). For example, a green apple in a basket full of red apples is salient because its color is distinct from the neighbors. In other words, a salient item contains something that its neighbors lack or *vice versa*. Such items can be a group of similar smaller salient parts which are perceived coherent due to perceptual grouping (Sporns et al., 1991; Alais et al., 1998). There is a direct relationship between this discrepancy and the amount of saliency. In addition to increasing the dissimilarity of the target and distractors, increasing the similarity among distractors themselves can enhance saliency (Scialfa et al., 1998; Phillips et al., 2006; Alexander and Zelinsky, 2012; Zehetleitner et al., 2013; Neokleous et al., 2016). What are the exact features that play a role to make a particular part of a scene salient in comparison to the other parts?

The answer depends on how much time passes while receiving the visual information. The more time spent scanning, the more complex features (physical factors), will affect saliency detection since more regions in the brain will be engaged. It is commonly accepted that attention is divided into top-down and bottom-up mechanisms. Top-down attention is attending to a certain object or feature that is goal-driven and voluntary. In this case, there is enough time for visual information to travel to areas higher than early visual processing parts in the brain (Wykowska and Schubö, 2010). As a result, higher level features such as shape and semantic categories [e.g., face and animals (Wolf and Horowitz, 2004)], as well as cognitive signals such as memory (Santangelo, 2015; Santangelo et al., 2015) or expectation (Itti and Baldi, 2009) are used. By contrast, bottom-up attention is considered involuntary and stimulus-driven. In other words, in bottom-up attention, a salient part of a scene captures attention immediately after looking (Pinto et al., 2013). The more salient a part of the visual scene is, the less top-down processing will be involved (Hodsoll et al., 2006). During brain development, the human visual system becomes more sensitive to the salient parts of the visual scene. Infants in the first year of life are sensitive to the most salient part of an image and children between 3 and 4 years and adults detect both the most salient object and the less salient ones (Sireteanu et al., 2005). Early visual regions in the brain such as primary visual cortex (Zhang et al., 2012) are responsible for handling the bottom-up attention mechanism. Therefore, features that are coded in this area or earlier (White et al., 2017) such as luminance contrast, color, motion, orientation, and size (including length and spatial frequency) are expected to guide bottom-up attention (Wolf and Horowitz, 2004). Investigating both high- and low-level features and their impact on saliency can help us understand the functionality of visual parts of the brain, model its function and predict responses to a new unseen image which is useful for applications such as advertising and brain modeling. But, low-level features constitute the building blocks of higher level features, before getting to the higher areas of the brain where semantics also matters. Extraction of these features is the basis of the models proposed for attention. Some of these models were inspired by feature integration theory (FIT) (Treisman and Gelade, 1980) and aimed to explain the functionality of the brain in detecting the most salient parts of a scene (Itti and Koch, 2001).

**FIGURE 1:**
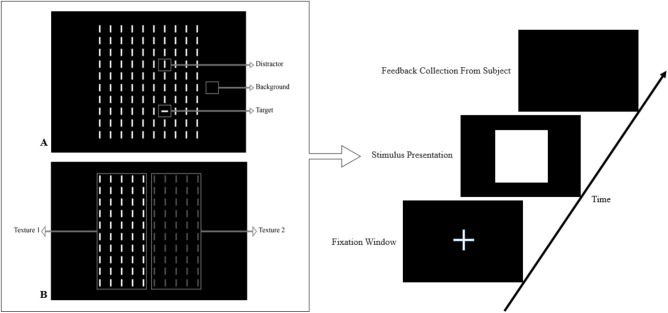
Template of common psychophysical experiments. The right image shows the timeline. It starts with presentation of a fixation point, continues with stimulus presentation, and stops after collecting behavioral responses from subjects. The task can be visual pop-out or visual search by presenting stimulus **(A)** or texture segmentation by presenting stimulus **(B)** in the left part of the figure.

In this paper, we review the studies that focused on low-level visual features in terms of saliency using psychophysical experiments. The focus is on the 2D psychophysical tasks that are designed to answer attention-related questions. The psychophysical experiments are usually designed by showing a sequence of images to subjects and receiving their behavioral responses (Figure 1). As the goal of these experiments is investigating the saliency of visual features, subjects are usually shown an image with zero, one or more salient parts. Then, various questions concerning the exact goal of the experiments are asked. Most of the studies use unconscious visual pop-out, voluntary visual search, or texture segmentation tasks. Visual pop-out happens just briefly after we look at a scene. The image mainly contains one target and a group of distractors that are placed in the background. The salient part immediately pops out and feedback from the subjects will be analyzed. The visual search starts with introducing the target to subjects. Then, the subjects will be instructed to search in a picture and report if they find the target as soon as possible. In texture segmentation tasks, one or a composition of multiple features is used to create an image with two or more textures. In these types of tasks, the subjects are instructed to respond immediately to the stimulus. We classified experiments in the studies reviewed in this paper into Tables 1–3 related to visual pop-out, visual search, and texture segmentation, respectively. The task details are given in each table. We excluded studies related to the investigation of the impact of higher level features on saliency (see Santangelo, 2015 and Baluch and Itti, 2011 for investigating the impact of high-level features on saliency), so tasks such as free-viewing will be omitted in this paper. Behavior registered during a free viewing task is affected by further processing in higher brain areas and feedback paths that are also activated. In these tasks, an image (e.g., a natural image) is displayed in front of the subject and one is asked to view it without defining any goal for several seconds. An eye tracker is usually used to record eye fixations during experiments. In some cases, subjects are instructed to explicitly declare which part is more salient (Borji et al., 2013) or they are asked some indirect but goal-driven questions (Tajima and Komine, 2015). Analyzing the results from these types of experiments has revealed other effective factors in saliency like faces or houses that are reported to be attractive (Treue, 2003).

**Table 1:** An overview of common visual pop-out experiments.

Task properties	Inference	Sample scheme	Reference
**B:** solid black (or white) **D:** array of parallel oriented white (or black) lines **T:** oriented line **Diff:** one or two features from orientation, color, intensity, direction of motion, density	Multiple features are added (linearly or non-linearly) in saliency.	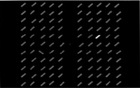	Nothdurft, 2000a (similar to Nothdurft, 2000b,c, 2002; Casco et al., 2006; Koene and Zhaoping, 2007; Zhaoping, 2008; Donk and Soesman, 2010; Zehetleitner et al., 2011; Krummenacher et al., 2014)

**B:** solid white **D:** vertical black rectangles **T:** vertical black rectangles **Diff:** length, width, or both	Detecting saliency due to length or width feature contrast takes more time in comparison with saliency due to superposition of them. Those features are added in Euclidean manner.	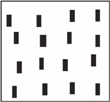	Pramod and Arun, 2014

**B:** squares with grating pattern **D:** not used **T:** square with grating pattern **Diff:** orientation	Saliency is encoded in monocular level.	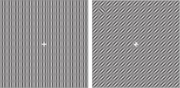	Stuit et al., 2010

**B:** solid gray **D:** parallel oriented lines surrounded by a frame **T:** oriented black lines **Diff:** orientation of target and frame lines	Left and right borders as well as full frame have more interaction in saliency detection than top and bottom borders.	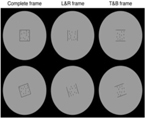	May and Zhaoping, 2009


*B, D, T, and Diff stand for background, distractors, target, and difference between target and distractors, respectively.*

**Table 2:** An overview of common visual search experiments.

Task properties	Inference	Sample scheme	Reference
**B:** solid white **D:** colored oriented lines **T:** colored or (and) oriented line **Diff:** orientation or (and) color	Binding happens in early stages of visual processing. But the relationship between features are represented in lateral areas.	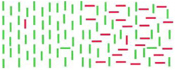	Wolfe and Cave, 1999

**B:** solid black **D:** gray-oriented lines with wave-like homogenous pattern **T:** oriented line **Diff:** orientation, intensity, or both	Contrast in feature of target and its local background induces saliency.	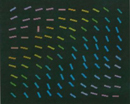	Nothdurft, 1993

**B:** solid black **D:** gray squares **T:** gray square **Diff:** size, intensity, or both	Size and intensity are functionally related.	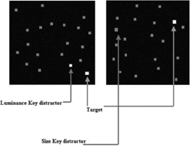	Huang and Pashler, 2005

**B:** solid black **D:** regular vertical colored line array (a column of lines are more salient than others in color or intensity) **T:** vertical broken line **Diff:** intensity or color with respect to the background except for the column in which the target is placed	It is easier to find the target on the part of image that is salient due to color and intensity but not orientation.	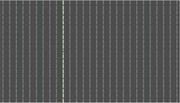	Jingling et al., 2013

**B:** grating **D:** circular gratings **T:** circular grating **Diff:** intensity, spatial frequency, and orientation	Background properties (in addition to distractors) affect saliency.	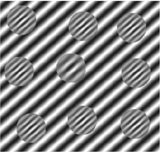	De Vries et al., 2013

**B:** surface texture **D:** not used **T:** small circle **Diff:** smoothness	Proposing surface texture to study low-level features in attention	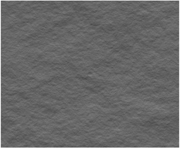	Clarke et al., 2008

**B:** pixels with random color from two colors (R-G or B-Y) **D:** not used **T:** solid squares **Diff:** color which is chosen from one of two colors used in the background	Yellowish targets in bluish background are more salient than other combinations.	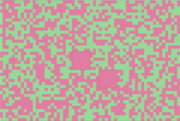	Wool et al., 2015

**B:** solid gray **D:** not used **T:** colored circles with various intensity **Diff:** hue and intensity	Blue is the least salient color in gray background and needs more fixations to be detected.	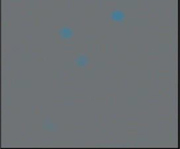	Etchebehere and Fedorovskaya, 2017

**B:** solid gray **D:** colored bars with various frequency **T:** colored bars with various frequency **Diff:** orientation and frequency	Orientation attracts more attention than spatial frequency.	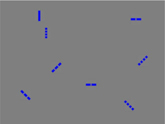	Phillips et al., 2006


*B, D, T, and Diff stand for background, distractors, target, and difference between target and distractors, respectively.*

**Table 3:** An overview of common texture segmentation experiments.

Task properties	Inference	Sample scheme	Reference
**B:** solid black (white) **D:** oriented (randomly) colored lines with a predefined texture **T:** not used **Diff:** not defined	Neurons in primary visual cortex answer to saliency of preferred feature rather than the feature itself.	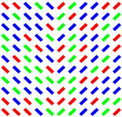	Jingling and Zhaoping, 2006 Zhaoping and May, 2007

**B:** basket texture background (textures are different in spatial frequency, orientation or both) **D:** not used **T:** not used **Diff:** not defined	Texture segmentation is easier considering contrast in multiple features in comparison with contrast in only one feature.	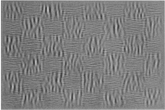	Bach et al., 2000

**B:** solid white **D:** black dots (a group of them were smaller or larger than others) **T:** not used **Diff:** not defined	Infants in the first year are able to detect saliency in high degree. In third and fourth years of life, they are sensitive enough to get lower degree of saliency as well.	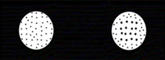	Sireteanu et al., 2005


*B, D, T, and Diff stand for background, distractors, target, and difference between target and distractors, respectively.*

The subjects are examined beforehand, to have normal or corrected to normal vision. In some studies, they must pass additional tests such as tests for color blindness. Analyzing the feedback of the subjects leads to understanding the function of the brain during the experiments. The response and reaction time of subjects are commonly extracted and further analyzed. It is believed that the saliency map, a map of visual scene which assigns higher values to more salient parts, is general and motor-unspecific, i.e., it affects several motor systems such as pointing movement as well as eye movements (Zehetleitner et al., 2011). The oculomotor system uses it to make the eyes focus on the salient part of the image (Treue, 2003; De Vries et al., 2017). In some cases, the eye movements of the subjects are also recorded to extract information from their conscious and unconscious reactions while looking at the screen.

In the following review, we divide feature saliency studies into five categories according to their objectives: Part I focuses on saliency produced by contrast in multiple features and includes investigations of the binding problem. In Part II, temporal and spatial properties of saliency are reviewed. Some studies investigated saliency in color domain which will be examined in Part III. In Part IV, the effects of background and border on saliency are considered. Finally, studies related to binocular rivalry are addressed in Part V. Finally, we conclude by discussing additional important points on saliency detection.

### Saliency in Multiple Features and the Binding Problem

Any feature difference between the object and its neighbors can make it salient. That is true for orientation and motion features as well as color, luminance intensity, etc. Comparing those features together has shown that saliency from color and luminance intensity captures our attention easier and faster (Nothdurft, 2000b). This is in line with the physiology of our eyes since the photoreceptors and ganglion cells in retina perceive color and luminance contrast, while other features are processed in higher level areas [e.g., primary visual cortex (V1 area) for orientation].

A method of comparing the saliency of two features in different contexts is presenting them as a key target and a key distractor within homogenous distractors and asking the subjects to uncover the salient target from the distractors. It has been shown that the less salient target requires considerably longer search time while the target with more saliency takes shorter response time to be uncovered from the key and other distractors. Huang and Pashler (2005) compared the object saliency based on its luminance and size disparity against a homogenous background (Table 2). In this visual search task, subjects were expected to distinguish a predefined target (which was brighter and larger than the background items) from a key distractor (that was either brighter or larger than the background items). The saliency was determined by the ratio between the target and background feature values. The results demonstrated that as the object and background difference in luminance or size increased, the target became more salient. Saliency was increased with increasing luminance at almost half the rate that it did with increasing size demonstrating that the luminance and size are functionally related (Huang and Pashler, 2005). As there is a relationship between size and spatial frequency (Hughes et al., 1996; Fiser et al., 2001), one might use the same method to investigate whether the same result will be concluded by comparing spatial frequency and luminance features. This approach is suitable for comparing the saliency of the two features; since the subjects are faced with both features simultaneously in their field of view. It is, however, important to put both features at the same distance and same visual angle from the fixation point. Otherwise, the result might be misleading as the subjects can be distracted by a feature just because it is easier (nearer to fixation point) to make saccade and not necessarily due to its saliency.

The saliency of an object is larger when the difference between the object and distractor is in more than one feature (Nothdurft, 2000a; Huang and Pashler, 2005; Krummenacher et al., 2014). That is true for pop-out, visual search, and texture segmentation (Bach et al., 2000). Surprisingly, adding a single feature to both target and distractors which already have differences in features, can increase the target’s saliency. Casco and his colleagues reported that although a near vertically oriented line (less than 45 degree of inclination) does not seem highly salient in a background with multiple 45° oriented lines, adding motion with the same velocity to all of the lines makes the near vertical lines more salient (Casco et al., 2006). This result is interesting since it shows that adding dynamics to the static orientation features makes them more salient. However, this was not true when they considered a more horizontal line as target. This extends the question on what happens with other features. One might investigate whether spatial frequency and orientation, for example, can have a similar effect as motion.

Neurons and populations of neurons in V1 are selective and tuned to particular features such as specific orientations, spatial frequencies, sizes, temporal frequencies, luminance contrast, and direction of movement (Jin et al., 2011; Lashgari et al., 2012; Jansen et al., 2015; Li et al., 2015). In order to detect saliency of a target that is in contrast with distractors in multiple features, the brain needs to integrate single features. Integration of information happens in the different levels of the brain. For example, assigning both tall and having black hair to a person is an example of integration in higher level processing areas. In the case of merging early visual features, this is done unconsciously (Keizer et al., 2015) and there is no preference for a particular feature to be bound more easily than others (Ward and Arend, 2012). How these features are combined is known as the binding problem (Wolfe and Cave, 1999).

According to FIT, different low-level features are extracted independently in parallel and make several single feature saliency maps. Then, they are combined to introduce the most salient object in the scene. Therefore, detecting saliency from one feature is supposed to take less time than multiple features. This hypothesis was examined by several studies. Pramod and Arun (2014) used an irregular line array with a target that is different in length, width, or both from the distractors for investigating the effect of individual features and their superposition. They concluded that integral features such as width and length of a rectangle combine in Euclidean manner while orientation and size, as two separable features in visual search, combine linearly. A saliency map that is an integration of a single feature is feature unspecific. That means in the integrated map, a particular object is salient but we do not know in what aspect and what is the exact feature that made that item salient. To investigate this, Krummenacher et al. presented a line array in a circular manner to the subjects and asked them to press a key if the target is present in the image as quickly and accurately as possible. The target was in contrast with distractors in terms of color, orientation, or both. Both behavioral and event-related potential (ERP) responses leaded the authors to suggest that both search and focal attentional selection are derived by salience-based and not feature-based signals (Krummenacher et al., 2014).

However, there is also evidence against FIT (Nothdurft, 2000a; Koene and Zhaoping, 2007). For example, Nothdurft (2000a) showed that the targets which are different in two features are more salient than the targets with just one feature but the saliency is not added linearly. They used a bar with different orientation, direction of motion, color, or a combination of them with respect to distractors as a test target. Furthermore, another bar with variable intensity values was defined as a reference target. The test target was placed randomly in the right or left half of the array and the reference target in the other half (Table 1). The image was presented for 150 ms and then masked with a cross array in which the test target, reference target, and distractors were not discriminable anymore. The subjects were asked to press one of two keys to specify which half was more salient. The experiment was repeated several times while changing the intensity of the reference target to quantify the amount of saliency. When the test target is perceived as salient as reference target, the intensity value of the reference target is interpreted as a saliency value. In an analogous study (Koene and Zhaoping, 2007), the authors presented an irregular line array with a randomly placed target to subjects. The result was similar to the previous study (Nothdurft, 2000a). They both support the hypothesis of creating one saliency map in primary visual cortex (Zhang et al., 2012).

Although there are different studies that focus on binding low-level features, there are still some gaps. Spatial frequency and phase as two low-level features are rarely investigated and questions such as how they integrate or interfere with other features are still unanswered. Phillips et al. (2006) reported that the spatial frequency dimension has less priority to be attended than orientation but the way they represented spatial frequencies (Table 2) differed from the conventional definition. They used a bar to represent an orientation and considered spatial frequency within the line by breaking it into multiple segments. The number of segments was considered as spatial frequency. By contrast, it is common to define spatial frequency as the number of cycles in a Gabor grating (De Valois et al., 1982; Cannon and Fullenkamp, 1991). Moreover, a recent study demonstrating a columnar organization of spatial phase in V1, has shown the visual space of objects is not only processed by spatial location, but also by absolute phase (Wang et al., 2015). Therefore, it would be very interesting to know how the spatial phases of the target and distractors can modulate the saliency of objects.

A different approach for studying the binding problem is investigating misbinding. Most of the existing studies in this area presented multiple visual features simultaneously to the subject, but there are neurons sensitive to multiple dimensions. So, it is hard to determine whether the result was due to the binding or the response of those neurons. Zhang et al. (2014, 2016) used fMRI and ERP brain imaging techniques to study neural grounding of color-motion misbinding, while presenting moving red and green dots on a black screen to the subject. Results show that misbinding and correct binding both happen in primary visual cortex but in a different hierarchy. It is reported that the conjunction of color and spatial frequency features happen in the right temporo-parietal junction (Pollmann et al., 2014) which makes it responsible for creating feature maps. The second study is in line with the former on assigning the responsibility of saliency map creation to parietal cortex (Gottlieb, 2007). It is believed that the motion system does not contain color sensitive neurons. The segregation of neural coding for color and motion was investigated by Cheadle and Zeki (2011). They designed a visual masking experiment to measure the interference of these two features (Cheadle and Zeki, 2011). In this experiment, the target contained both motion and color features (green and yellow) while the mask contained only one of them (for the color mask just red and blue and for the motion mask a white moving circle were considered). Subjects were instructed to report both the color and motion direction of the target. The authors found that color and motion impaired the judgment of these features, respectively. This is reported as evidence for separation of color and motion in the visual system.

The saliency of an object which is different in multiple features from its background is supposed to be more than single feature contrast. It is worth mentioning that assigning different features to parts of an image in particular manners makes features interfere. This leads to increasing distractor–distractor similarity and impairs saliency detection based on one feature. For color and orientation interference in a texture segmentation task, consider two textures of regular oriented bars, each with different orientation that are shown to subjects. A few predetermined colors are assigned to each bar randomly. Segmentation of this texture in terms of orientation (with a unique color) is easy. But as Jingling and Zhaoping (2006) assigned more colors randomly to the bars, the task became harder because of interference of color in orientation (Table 3). Using an eye tracker is useful in these studies as it assures the researcher that what is reported by the subjects is matched with what they have seen.

Overall, difference between an object and its neighbors in one or more dimensions leads to saliency. Adding a single feature such as motion to both target and distractors which have already had differences in another feature such as orientation can increase the discrepancy between them and facilitates saliency detection in some situations. The effect of features other than motion in similar situation has not been studied yet. On the other hand, adding a feature such as a different color to distractors reduces their similarity and impairs detecting the salient item. Which means that the saliency map is made after binding different dimensions. Some studies support the idea of FIT in building single feature-specific maps and combining them to make the feature-unspecific master saliency map. While others advocate the theory of a single saliency map in primary visual cortex that rejects independent contribution of features. There is no consensus on how the brain binds low-level features and what parts are responsible for this binding. Comparing saliency of various features together is useful in predicting human attention to the saliency area. Therefore, implementing different features in the same image at the same eccentricity with respect to the initial fixation would be very important. Then, the subject will be able to make a saccade to all of them. Recording gaze positions of the eyes is therefore essential to understand where involuntary attention is concentrated. In the next section, we will review how saliency detection can be affected by the other features such as temporal and spatial properties.

### Temporal and Spatial Properties of Saliency

Saliency is detected immediately when we look at a scene in the early processing area of the visual cortex. After that, information travels to higher areas and further processing works to identify the complex objects and the details of the visual scene (Nothdurft, 2002). How long is the time course for capturing the salient feature properties? A research study has simultaneously compared the response time of the saliency effect of two competitive targets which were presented against the regular orientation lines in a pattern on the background (Nothdurft, 2000b). In this visual task, a test and a reference bar target were randomly placed on each side of the fixation point (the left or right of the visual field). The test target was brighter than the reference one and they were, respectively, orthogonal to and parallel with the surrounding bars. The subjects were asked to report which of the two targets was more salient (Nothdurft, 2000b; Table 1). The experiments have shown that the orientation discrepancy takes longer to detect than luminance contrast. The results also demonstrated that the saliency increases as presentation time increases up to about 100–150 ms. After that time, the saliency did not change anymore. It is well known that the timing of responses in visual cortex is a key feature to distinguish between bottom-up and top-down attention (Liu et al., 2009). The results are in line with the study that suggested backward connections are added after about 200 ms (Garrido et al., 2007).

Detecting saliency in different features does not follow the same time course. Color and contrast luminance have higher temporal resolution than other features. Nothdurft (2000b) has reported this after presenting an irregular line array with one target among distractors with different flicker frequency to the subjects. When the replacement time was too short, the subjects were unable to see the salient bar. But color and luminance needed less replacement time to be detected in comparison to other features. This is in line with the findings that assign the responsibility of detecting color and intensity to retina and lateral geniculate nucleus (LGN) and other features to cortical areas since it takes less time for the information to get to those areas and then travel to the visual cortex.

A scene usually has several objects, each one with a different saliency respective the background. People tend to get the most salient information in shorter time courses and have shorter response time, while the less salient object remains in mind for longer periods of time. In fact, the difference between the most and the least salient objects in the scene does not appear in saliency map; in contrary, they are different in the time at which they are detected. The most salient object is detected first and the least salient one afterward (Dombrowe et al., 2010). Donk and Soesman (2010) designed a regular line array with two targets, one was salient with respect to the distractors, whereas the other one was inconspicuous. The stimulus was presented in 42, 158, or 483 ms and after that a probe was presented. The subjects were required to locate the probe while keeping fixation point at the screen center. The response time was recorded and analyzed. They found that salience information just remains shortly in the brain and subsequently, and it will be modified by more exact information about the object like its category. It is thought that temporal properties of saliency due to not only low-level features but also high-level ones are useful in advertising applications in real world.

Spatial properties of saliency evoked by different features are taken under consideration as well (Nothdurft, 2000b,c; Töllner et al., 2011). Nothdurft (2000c) reported that orientation and motion saliency are more detectable in medium density rather than low and high densities. This was postulated after presenting a regular line array in which the density of lines in the left half was different from the right one while asking the subjects to specify the most salient part. On the other hand, removing all distractors and putting the object on a blank background makes the object more salient than other situations. Nothdurft (2000b) showed that the saliency of a single line in one half was more than a test target among distractors in another half. This result is also against FIT, since according to this theory, both sides should have the same saliency.

As mentioned above, several studies have shown that different features provide different time resolutions in saliency detection. Color and luminance contrast have less time course in comparison with other features such as orientation (Nothdurft, 2000b). This might be due to their detectability in early stages of visual information processing in retina and LGN. It is commonly believed that the most salient part of a scene does not remain in the brain for long time. In fact, as the time passes by, more detailed and higher level information will be involved and the most salient part will be replaced rapidly with the less salient parts (Dombrowe et al., 2010; Donk and Soesman, 2010). Different features have different spatial resolutions as well. For example, saliency due to differences in orientation and motion is detected in medium density more easily than low or high density (Nothdurft, 2000c). Time and spatial resolutions due to differences in multiple features have not yet been studied. Although analyzing behavioral data helps us to understand more about temporal properties of saliency, an eye tracker is able to record both conscious and unconscious saccades from subject’s eye movements. It is useful to understand how many of the saccades, especially the first ones, are voluntary. Is the first saccade always toward the most salient part of the scene? As mentioned before, the temporal resolution of color and luminance contrast (Komban et al., 2014) is higher than other feature properties. Can we expect similar results from infant behaviors? What is the effect of age on the minimum understandable temporal and spatial resolution of various low-level features? Understanding more about temporal and spatial resolutions of saliency will be very useful in attracting the gaze and attention of the observers on the advertised items (Lee and Ahn, 2012). In a very small amount of time, low-level features matter more and are important in advertising, for example, for visitors making decisions to choose certain features. In such high-level applications, understanding how the temporal resolution of saliency affects human memory is also important. How much time is enough for presenting an advertisement to be sure that viewer’s attention is attracted to the salient part of the scene in order to memorize it?

### Saliency in Color and Luminance

Attention can be automatically captured by objects that are salient in color and luminance (Turatto and Galfano, 2000, 2001). Main colors which are considered in studies are blue, red, green, and yellow (Mullen, 2002). A yellowish target is more salient than a bluish target. But the saliency of red and green is the same. These relationships were reported by Wool et al. (2015) after investigating the saliency of unique hues. They presented an image with very small squares of two predefined colors that are randomly placed on a background with several larger squares with one of those two colors as targets (Table 2). The subjects were asked to specify the number of targets in the image. The shorter response time showed that a certain target color was more salient. Poirier et al. (2008) designed a different dynamically adjusting experiment to compare the saliency of red and green. The image was a randomly colored green and red texture. The intensity of each part of the image was also assigned randomly. The subjects hold one of the mouse buttons to increase or the other one to decrease the intensity and make the image homogeneous in the case of saliency. People tend to decrease the intensity of more salient parts of image in order to make them as salient as other parts. The above two studies considered color opponency in their task design which led to examining blue vs. yellow and green vs. red but it remains unclear how these two groups interact with each other? Is green as salient as red in a blue background?

Comparing the main colors in a gray background shows that blue is the least salient one. Etchebehere and Fedorovskaya (2017) put several circles with specific colors in CIALAB space but with different intensity values in a gray background (Table 2). They asked subjects to report the number of circles. Subjects found the circles with higher chroma easier with no fixation while for lower chroma circles they needed to fixate. This is because color and luminance intensity (compared to features such as orientation) can be captured covertly, with less need to fixate. According to the analysis of subjects’ eye tracking information and their response time, as a hue gets more attention, it needs fewer fixations. The maximum number of fixations happened for blue targets.

In the case of luminance contrast intensity, Pashler et al. (2004) investigated the default tendency of attending to high-contrast items. They showed a black background with tens of randomly placed digits which were colored with a high or low intensity green color. The subjects were asked to press two keys whether the target is “8” or “9.” The authors reported that people were able to successfully ignore high-luminance contrast irrelevant items and attend to a low-luminance contrast target. This result demonstrates that people can attend to different levels of luminance contrast.

Overall, considering different color hues, blue is commonly reported as the least and yellow as the most salient color (Wool et al., 2015; Etchebehere and Fedorovskaya, 2017). Using an eye tracker does not seem to be as useful, since color can be perceived covertly. Nevertheless, it has been beneficial in some cases (Etchebehere and Fedorovskaya, 2017). As the receptive field of color selective neurons has the shape of center-surround, the background is important and needs to be considered in future studies. Luminance contrast and color contrast are perceived in a very small amount of time just after projection of the image on the retina which highlights the importance of these two features for visually understanding the environment. For example, luminance provides information about the time and whether it is day or night. Color also can affect our visual perception of the environment. Is there any relationship between subjects’ favorite color and how they perceive it in early visual areas? How are color and luminance saliency different in normal and color-blinded subjects? How does the color background in different saliency tasks influence the saliency map?

### Effect of Background and Borders on Saliency

Any contrast between physical factors of an item and its neighbors or background makes it salient. This contrast can be influenced by high-level features of an image which is detectable after involving higher level visual processing areas in the brain. Before that, low-level feature discrepancy can make an object salient (Nothdurft, 2000a). Nothdurft (1993) examined this by performing an experiment in which subjects were instructed to report the presence or absence of a vertical line in an irregular array. Distractors in this array followed a wave-like pattern and the target (vertical bar) was placed in different parts of this pattern (Table 2). They found that the target detection was easier when its orientation was more contrasted with its neighboring distractors. In fact, the local feature contrast is important to detect salient objects. Local dissimilarity also facilitated texture segmentation and grouping in a part of image (Nothdurft, 1992).

The salient part of an image attracts attention, therefore visual search is facilitated when the target is placed in the salient part. This was supported by Jingling et al. (2013) who designed an experimental task with a multi-column line array background, one of which was more salient than the others (Table 2). Subjects were instructed to find a broken line that was placed in the salient column in some trials and in other parts of the array in other trials. The authors found that visual search is easier when the target is shown on the column which is salient in terms of luminance intensity and color contrast but not orientation (it impairs visual search task). On the other hand, global saliency impairs the search for a local element. Adding saliency to a huge part of the image reduces the accuracy of saliency detection whether the target is situated in the salient or non-salient parts (Jingling and Tseng, 2013).

V1 neurons respond weaker to their preferred feature when it is surrounded by the same feature. But, when its surrounding is different, the response is stronger. That is the V1 neuron’s answer to the saliency of the preferred feature rather than the feature itself (Li, 2002). This was supported by Zhaoping and May (2007) in a texture segmentation task composed of two bar arrays with different orientation. They reported that the response to the bars on the border of two-line arrays with the same orientation is stronger than when the lines are placed within them.

Borders of images are also important in visual perception. May and Zhaoping (2009) have shown the effect of borders in orientation pop-out task. They presented images with an irregular line array within a square frame and recorded the subjects’ response time in answering whether the target is present or absent. In some trials, they abolished the left and right lines of the frame and in the other trials the top and bottom lines were removed instead (Table 1). Furthermore, in some trials, the frame was rotated. They demonstrated that the saliency effects of left and right borders are the same as the full frame case but stronger than top and bottom borders. Furthermore, the response time of tilted target in vertical frames was less than vertical target in tilted or vertical frame (but tilted distractors). They argued that there are two reasons why saliency is affected by frame. The first is due to providing a frame of reference by a high-level configural cue and the second is because of iso-orientation competition arisen from the parts of the frame parallel to the target and distractors that are parallel to it.

Most of the experiments that were mentioned until now considered distractors as the background while distractors were in fact only part of the background. The properties of the background itself have a large impact on the search time. This was reported by De Vries et al. (2013) after using circles with the texture of grating as targets and distractors and putting them all on a background again with grating texture. Background, target, and distractors were different in orientation, spatial frequency and luminance (Table 2). In another study on the importance of background features, Clarke et al. (2008) proposed a surface texture (Table 2) to be used in visual search experiments. They showed that the roughness of the surface can be changed to vary the difficulty, e.g., with increasing the roughness of stimuli the subjects’ response time will increase. It is equivalent to changing the set size in the common line array visual search tasks. Depth or luminance contrast of the background also plays a role on the efficiency of the task.

In brief, it is commonly accepted that the dissimilarity between an object and its surrounding items makes it salient (Nothdurft, 1992, 1993; Jingling and Tseng, 2013). However, decreasing the similarity among the surrounding items themselves can impair saliency detection (Jingling and Tseng, 2013). It is even suggested that V1 neurons respond to the saliency of their preferred feature and not the feature itself (Li, 2002; Zhaoping and May, 2007). Therefore, considering meaningful backgrounds for a task is also important especially because we know that some neurons in early visual processing areas benefit from center-surround receptive fields. This receptive field is sometimes small and does not contain distractors. Assigning distracting features to the background is a better strategy. To the best of our knowledge, De Vries et al. (2013) did the only study which considered gratings (for targets, distractors, and background) into account. Ignoring distractors in this task made it similar to the classical line array tasks. One might investigate to see, whether considering this new configuration changes the results which were already reported by the line array experiments. The effect of background is also important to be considered in search asymmetry (Rosenholtz, 2001). It is also worth investigating to see whether the salient targets are greatly affected either by background or distractors at the same visual task. Although the effect of borders has been shown by May and Zhaoping (2009), almost all of the designed psychophysical tasks are borderless. Changing the properties of a border feature can be an easy way to modulate the saliency of an object in applications such as advertising.

### Saliency in Binocular Rivalry

The eyes compete when an image is presented to one eye and another different image to the other eye. It means that instead of perceiving the superposition of two images, one of them is perceived at each moment. This is called binocular rivalry and can guide attention (Zou et al., 2017). This fact is useful to study monocular vision and to compare it with binocular vision. Visual processing starts separately within each eye. Then, the information travels to lateral areas where the input from both eyes is combined. Studies in various dichoptic conditions showed that ocular singleton competes effectively in attention. Monocular neurons are rare in cortical areas other than V1 and this supports the theory which favors creation of a bottom-up saliency map in primary visual cortex (Zhaoping, 2008). Another evidence for this theory was reported by Stuit et al. (2010) who performed a pop-out visual task (Table 1) to see if saliency in a suppressed image can bias how the observer tends to scan the image. The authors presented two different images of a grid of Gabor stimuli on a gray background to different eyes. In one image, the grating stimuli had similar properties and there is no salient item there considered as a suppressor image. In the other one, the grating stimuli had similar properties except for one of them which was different in orientation and considered as a test image. The suppressor image was shown at full luminance contrast at the beginning of each trial but the luminance contrast of the test image was set to 0% at first so it was invisible at the start of a trial. The luminance contrast of the test image increased gradually to 100% over a period of about 10 s. Subjects were instructed to press a key quickly after perceiving the test image and move the cursor to the place where they perceived this alternation and click on that location. The results demonstrated that saliency is unconsciously detected at the monocular level.

In summary, studies have demonstrated that saliency is detected at the monocular level (Zhaoping, 2008; Stuit et al., 2010). Understanding the details of how the eyes compete on perceiving visual information can help to answer the question of whether any preference for each eye to detect saliency is due to specific features in comparison with the other eye. How do they compete when two images with different salient parts are presented to each eye separately? What if we show salient items with different temporal resolution, for example, an image with orientation contrast to one eye and an image with color contrast to another eye? How does information from the eyes bind together in this case? Can saliency detection at the monocular level affect which eye will be dominant? Using an eye tracker to record the subject’s eye movements during similar experiments is useful to analyze unconscious reaction of the eyes after presenting different images to each eye.

## Discussion

Our visual system interacts with the properties of objects that surround us to determine which part of the visual scene will be sequentially selected for processing. Electrophysiological and neuroimaging studies in primates suggest that multiple stimuli are not processed independently, but compete and interact with each other in a mutually suppressive way (Desimone and Duncan, 1995; Duncan and Nimmo-Smith, 1996; Beck and Kastner, 2009). Moreover, the competitive interactions among multiple stimuli for neural representation occur simultaneously and operate in parallel across cortical brain areas. Overall, visual attention can resolve the competition by filtering out the information and reducing the suppressive influences of nearby stimuli, thereby enhancing information processing at the attended location (Desimone and Duncan, 1995; Kastner and Pinsk, 2004). The part that stands out more attracts more attention and has higher priority to process. At first glance, this part is salient considering low-level features. Low-level dissimilarity between an item and its neighbors and low-level similarity between the neighbors themselves are important to make an item locally salient. Sometimes adding an identical low-level feature to both object and its neighbors can increase the saliency of the target and make it easier to detect (Casco et al., 2006). On the contrary, adding a particular feature with various properties (e.g., color with different hue values) to both target and distractors randomly can degrade the performance of salient target detection (Jingling and Zhaoping, 2006). This is because different neurons which are selective to various features interact to detect a part of a scene as the most salient part. Each feature has different levels of contribution that can change from trial to trial (Wolfe, 1998). Although it is widely accepted that low-level features undoubtedly guide bottom-up attention (Wolf and Horowitz, 2004), there are still debates on how these feature dimensions interact together to make a master saliency map. The exact mechanism to bind these features in the early visual areas is still unknown. FIT (Treisman and Gelade, 1980), the most famous theory in this area, suggests that there is an independent saliency map for each feature dimension. However, the final saliency map is made by linear integration of all these maps and represents how much each part of a scene is salient (Pramod and Arun, 2014). Saliency computational models according to FIT were rather successful to predict the salient part of an image in most cases (Itti and Koch, 2001) where some contrary findings are also reported. For example, there is evidence reported by Nothdurft (2000a) and Koene and Zhaoping (2007) who showed that saliency due to multiple feature contrast leads to faster response time in comparison with single feature contrast. The response time for saliency detection due to multiple feature contrasts is shorter than the time for calculating all features in parallel and combining them. In FIT, by defining a saliency map for each feature separately, the response of neurons selective to more than one feature is ignored. However, they are able to detect saliency due to multiple feature contrasts easily. Another theory suggests that neurons in primary visual cortex respond to saliency of their preferred feature and not to the feature itself (Zhang et al., 2012). Accordingly, the response of a neuron selective to the 45° orientation is stronger when this feature is surrounded by 135° orientation than when it is surrounded by the same orientation. That is true for all V1 neurons whether they are selective to one feature or more. This puts emphasis on the importance of background on saliency detection (Mannion et al., 2017). On the contrary, almost all studies in this domain did not pay attention to this fact by considering solid black, gray, or white colors as a background in their visual tasks (Nothdurft, 1993, 2000a,b,c, 2002; Wolfe and Cave, 1999; Huang and Pashler, 2005; Koene and Zhaoping, 2007; Zhaoping, 2008; May and Zhaoping, 2009; Donk and Soesman, 2010; Zehetleitner et al., 2011; Krummenacher et al., 2014; Pramod and Arun, 2014).

Although we know the receptive field of orientation selective neurons follows a Gabor-like template, using bars still is common in literature to define an orientation. On the other hand, the impact of some important low-level features such as spatial frequency and phase has not been studied yet in psychophysical experiments. Using Gabor stimuli instead of lines as objects facilitates focusing on these features. One might design a task in this way and study how spatial frequency difference or phase difference leads to saliency and how this interferes with or binds to other low-level features and what the time course is. Borders also have a significant impact on saliency detection. It is reported that the saliency of a target among distractors can be affected by the orientation of their surrounding square frame (May and Zhaoping, 2009). It can change the frame of reference and makes the border orientation interact with the orientation of the bars inside it. Almost all studies ignored the effect of the surrounding frame in their experimental tasks (Nothdurft, 1993; Wolfe and Cave, 1999; Jingling and Zhaoping, 2006; Zhaoping and May, 2007; Jingling et al., 2013). Answers to remaining questions such as how other factors of borders, such as their shape and color, affect detection of the salient items within them, would be beneficial to know.

Our perceived visual information is first processed in the retina, LGN, and the primary visual cortex then spreads to higher cortical areas in order to integrate the details of visual scene and make sense of what we are observing. The low-level features such as color and luminance are encoded in the areas before cortical regions while the features such as orientation and spatial phase are detected in primary visual cortex. This is why subjects respond faster to saliency due to color or luminance contrasts (Nothdurft, 2000b). The fMRI and electrophysiological studies have demonstrated that neural activity in the primary visual cortex is responsible for creating the bottom-up saliency map of visual scene (Zhang et al., 2012; Chen et al., 2016). Studies related to binocular rivalry also support this idea since it has been reported that saliency is detected at the monocular level (Stuit et al., 2010). Neurons responsible for monocular processing are rare in areas other than primary visual cortex. In higher cortical areas, complex features are substantially recognized and, through combining feedforward and feedback signals as well as cortical and subcortical communications, can be meaningfully detected. This is why meaningful objects such as letters, shapes, or other high-level features can attract attention too (Fellrath et al., 2012; Pinto et al., 2013; Barras and Kerzel, 2017). Therefore, the low-level features are the fundamental structures of the salient objects. There is a common point between effective features (orientation, color, spatial frequency, etc.) in bottom-up saliency detection: all of them have some neurons in the brain dedicated to encode the features directly; let us call them basis neurons. To detect saliency of other features such as shapes, the visual input is parsed to be detectable by the basis neuron and their response travel hierarchically to higher visual cortical areas. This is why bottom-up attention is so fast but top-down needs more time to use both higher level features and cognitive signals with backward connections.

At first glance, the part of the scene with the highest amount of discrepancy with respect to its neighbors, in terms of low-level features, is what catches the eye. As time passes, less salient items become attractive (Liu et al., 2009). After a long time, as much as multiple hundreds seconds, visual processing is controlled and integrated by both bottom-up stimuli and top-down directed attention mechanisms (Kastner and Ungerleider, 2000; Reynolds and Chelazzi, 2004; Serences and Yantis, 2006; Beck and Kastner, 2009; Szczepanski et al., 2010). Concept also becomes important in this case (since high level features are extracted) and people tend to focus on meaningful objects which are salient. Therefore, the time course is an important point to predict what kind of information will be extracted. This is useful in advertising where a short but memorable advertisement is appreciated. There are studies concerning how effective use of different features can lead the audience’s attention to a specific part of an image or video in a given time scale (Rosbergen et al., 1997; Pieters and Wedel, 2004; Vijfvinkel, 2014). The background of visual tasks is also important and has a significant effect on saliency detection (Mannion et al., 2017). Almost all studies in this domain did not pay attention to this fact by considering the black or gray colors as a background in their visual tasks. Researchers in this area should be aware of the background impact during task design. Objects on the background also matter. Although we know the receptive field of selective orientation neurons follows a Gabor-like template, using bars still is common in literature to define an orientation. On the other hand, the impact of important low-level features such as spatial frequencies and spatial phases has not been studied yet. Using psychophysical experiments, grating stimuli instead of lines as objects facilitates focusing on these features. One might design a task in this way and show how difference in spatial frequency and phase leads to saliency and how this interferes with or binds to other low-level features and what is its time course is. It might also be interesting to study the impact of the higher level factors on saliency and answer questions such as how much personal experience on different concepts modulate saliency detection in natural images? Can we say saliency due to low-level features on most of the scenes is common between different subjects but high-level features on some special scenes might lead to subject differences in reporting salient regions?

## Conclusion

We reviewed studies related to saliency due to low-level features in visual tasks. Scientists in this domain agree on many aspects of early visual processing. They believe that a part of an image can be salient because of its difference from background in terms of one or more high-level or low-level features or similarity with the neighbors. The difference due to multiple features makes the target more salient than the difference provided by a single feature (Nothdurft, 2000a; Huang and Pashler, 2005; Krummenacher et al., 2014). Temporal and spatial resolutions have effects on detecting saliency of a target. It has been shown that temporal resolution of color and luminance is higher than other features (Nothdurft, 2000b) and a target is more salient in terms of orientation and motion in medium spatial resolution (Nothdurft, 2000b). Investigations in the color domain have shown that yellowish targets are more salient than the other three main colors: bluish, reddish, and greenish. Background and borders of the image significantly affect saliency and are important to be considered in future task design. Finally binocular rivalry studies suggested that saliency happens at the monocular level. Neurons responsible for monocular processing are rare in cortical areas other than V1. However, there are still debates on some aspects such as the way different features are integrated together and the part of the brain that is responsible for it.

There are many other unanswered questions that have been proposed in this review. Questions such as how different background and border properties can affect saliency detection? Do we get the same result after using Gabor stimuli instead of line arrays in the above mentioned saliency tasks? How low-level features such as spatial frequency and phase modulate saliency and interfere with or bind to other low-level features? Studying low-level features in terms of saliency is fundamental as they are the building blocks of higher level features such as object category and concepts which are understandable with the help of cognitive subsystems such as working memory (Santangelo and Macaluso, 2013; Pedale and Santangelo, 2015). In fact, what we attend to in each moment of our daily lives is the result of interaction of both bottom-up and top-down processes in the brain which recruits low-level features (e.g., orientation, luminance, and color contrast), high-level features (e.g., shape and object category), and endogenous signals (e.g., memory and expectation).

## Author Contributions

SK, HM, and RL contributed to the design and review of the articles and to the writing of the manuscript.

## Conflict of Interest Statement

The authors declare that the research was conducted in the absence of any commercial or financial relationships that could be construed as a potential conflict of interest. The reviewer TB and the handling Editor declared their shared affiliation at the time of the review.

## References

[B1] Alais D. Blake R. Lee S. H. (1998). Visual features that vary together over time group together over space. *Nat. Neurosci.* 1 160–164. 10.1038/414 10195133

[B2] Alexander R. G. Zelinsky G. J. (2012). Effects of part-based similarity on visual search: the Frankenbear experiment. *Vis. Res.* 54 20–30. 10.1016/j.visres.2011.12.004 22227607 PMC3345177

[B3] Bach M. Schmitt C. Quenzer T. Meigen T. Fahle M. (2000). Summation of texture segregation across orientation and spatial frequency: electrophysiological and psychophysical findings. *Vis. Res.* 40 3559–3566. 10.1016/S0042-6989(00)00195-4 11116160

[B4] Baluch F. Itti L. (2011). Mechanisms of top-down attention. *Trends Neurosci.* 34 210–224. 10.1016/j.tins.2011.02.003 21439656

[B5] Barras C. Kerzel D. (2017). Salient-but-irrelevant stimuli cause attentional capture in difficult, but attentional suppression in easy visual search. *Psychophysiology* 54 1826–1838. 10.1111/psyp.12962 28752665

[B6] Beck D. M. Kastner S. (2009). Top-down and bottom-up mechanisms in biasing competition in the human brain. *Vision Res.* 49 1154–1165. 10.1016/j.visres.2008.07.012 18694779 PMC2740806

[B7] Blake R. Lee S. H. (2005). The role of temporal structure in human vision. *Behav. Cogn. Neurosci. Rev.* 4 21–42. 10.1177/1534582305276839 15886401

[B8] Borji A. Parks D. Itti L. (2014). Complementary effects of gaze direction and early saliency in guiding fixations during free viewing. *J. Vis.* 14:3. 10.1167/14.13.3 25371549

[B9] Borji A. Sihite D. N. Itti L. (2013). What stands out in a scene? A study of human explicit saliency judgment. *Vis. Res.* 91 62–77. 10.1016/j.visres.2013.07.016 23954536

[B10] Cannon M. W. Fullenkamp S. C. (1991). Spatial interactions in apparent contrast: inhibitory effects among grating patterns of different spatial frequencies, spatial positions and orientations. *Vis. Res.* 31 1985–1998. 10.1016/0042-6989(91)90193-9 1771782

[B11] Carrasco M. (2011). Visual attention: the past 25 years. *Vis. Res.* 51 1484–1525. 10.1016/j.visres.2011.04.012 21549742 PMC3390154

[B12] Casco C. Grieco A. Giora E. Martinelli M. (2006). Saliency from orthogonal velocity component in texture segregation. *Vis. Res.* 46 1091–1098. 10.1016/j.visres.2005.09.032 16289199

[B13] Cheadle S. W. Zeki S. (2011). Masking within and across visual dimensions: psychophysical evidence for perceptual segregation of color and motion. *Vis. Neurosci.* 28 445–451. 10.1017/S0952523811000228 21835096 PMC3472342

[B14] Chen C. Zhang X. Wang Y. Zhou T. Fang F. (2016). Neural activities in V1 create the bottom-up saliency map of natural scenes. *Exp. Brain Res.* 234 1769–1780. 10.1007/s00221-016-4583-y 26879771

[B15] Clarke A. D. F. Green P. R. Chantler M. J. Emrith K. (2008). Visual search for a target against a 1/fβ continuous textured background. *Vis. Res.* 48 2193–2203. 10.1016/j.visres.2008.06.019 18639574

[B16] De Valois R. L. Albrecht D. G. Thorell L. G. (1982). Spatial frequency selectivity of cells in macaque visual cortex. *Vis. Res.* 22 545–559. 10.1016/0042-6989(82)90113-4 7112954

[B17] De Vries J. P. Hooge I. T. Wertheim A. H. Verstraten F. A. (2013). Background, an important factor in visual search. *Vis. Res.* 86 128–138. 10.1016/j.visres.2013.04.010 23623804

[B18] De Vries J. P. Van der Stigchel S. Hooge I. T. Verstraten F. A. (2017). The lifetime of salience extends beyond the initial saccade. *Perception* 47 125–142. 10.1177/0301006617735726 29183222

[B19] Desimone R. Duncan J. (1995). Neural mechanisms of selective visual attention. *Annu. Rev. Neurosci.* 18 193–222. 10.1146/annurev.ne.18.030195.001205 7605061

[B20] Dombrowe I. C. Olivers C. N. Donk M. (2010). The time course of color-and luminance-based salience effects. *Front. Psychol.* 1:189. 10.3389/fpsyg.2010.00189 21833249 PMC3153798

[B21] Donk M. Soesman L. (2010). Salience is only briefly represented: Evidence from probe-detection performance. *J. Exp. Psychol. Hum. Percept. Perform.* 36 286–302. 10.1037/a0017605 20364919

[B22] Duncan J. Nimmo-Smith I. (1996). Objects and attributes in divided attention: surface and boundary systems. *Percept. Psychophys.* 58 1076–1084. 10.3758/BF03206834 8920843

[B23] Etchebehere S. Fedorovskaya E. (2017). On the role of color in visual saliency. *Electronic Imaging* 2017 58–63. 10.2352/ISSN.2470-1173.2017.14.HVEI-119

[B24] Fellrath J. Blanche-Durbec V. Schnider A. Jacquemoud A. S. Ptak R. (2012). Visual search in spatial neglect studied with a preview paradigm. *Front. Hum. Neurosci.* 6:93. 10.3389/fnhum.2012.00093 22529795 PMC3328796

[B25] Fiser J. Subramaniam S. Biederman I. (2001). Size tuning in the absence of spatial frequency tuning in object recognition. *Vis. Res.* 41 1931–1950. 10.1016/S0042-6989(01)00062-1 11412885

[B26] Garrido M. I. Kilner J. M. Kiebel S. J. Friston K. J. (2007). Evoked brain responses are generated by feedback loops. *Proc. Natl. Acad. Sci. U.S.A.* 104 20961–20966. 10.1073/pnas.0706274105 18087046 PMC2409249

[B27] Gottlieb J. (2007). From thought to action: the parietal cortex as a bridge between perception, action, and cognition. *Neuron* 53 9–16. 10.1016/j.neuron.2006.12.009 17196526

[B28] Hodsoll J. P. Humphreys G. W. Braithwaite J. J. (2006). Dissociating the effects of similarity, salience, and top-down processes in search for linearly separable size targets. *Percept. Psychophys.* 68 558–570. 10.3758/BF03208758 16933421

[B29] Huang L. Pashler H. (2005). Quantifying object salience by equating distractor effects. *Vis. Res.* 45 1909–1920. 10.1016/j.visres.2005.01.013 15797780

[B30] Hughes H. C. Nozawa G. Kitterle F. (1996). Global precedence, spatial frequency channels, and the statistics of natural images. *J. Cogn. Neurosci.* 8 197–230. 10.1162/jocn.1996.8.3.197 23968149

[B31] Itti L. Baldi P. (2009). Bayesian surprise attracts human attention. *Vis. Res.* 49 1295–1306. 10.1016/j.visres.2008.09.007 18834898 PMC2782645

[B32] Itti L. Koch C. (2001). Computational modelling of visual attention. *Nat. Rev. Neurosci.* 2 194–203. 10.1038/35058500 11256080

[B33] Jansen M. Li X. Lashgari R. Kremkow J. Bereshpolova Y. Swadlow H. A. (2015). Chromatic and achromatic spatial resolution of local field potentials in awake cortex. *Cereb. Cortex* 25 3877–3893. 10.1093/cercor/bhu270 25416722 PMC4585519

[B34] Jin J. Wang Y. Lashgari R. Swadlow H. A. Alonso J. M. (2011). Faster thalamocortical processing for dark than light visual targets. *J. Neurosci.* 31 17471–17479. 10.1523/JNEUROSCI.2456-11.2011 22131408 PMC3470425

[B35] Jingling L. Tseng C. H. (2013). Collinearity impairs local element visual search. *J. Exp. Psychol. Hum. Percept. Perform.* 39 156–167. 10.1037/a0027325 22329767

[B36] Jingling L. Tseng C. H. Zhaoping L. (2013). Orientation is different: interaction between contour integration and feature contrasts in visual search. *J. Vis.* 13 26–26. 10.1167/13.3.26 24023276

[B37] Jingling L. Zhaoping L. (2006). A theory of a saliency map in primary visual cortex (V1) tested by psychophysics of color-orientation interference in texture segmentation. *Vis. Cogn.* 14 911–933. 10.1080/13506280500196035

[B38] Kastner S. Pinsk M. A. (2004). Visual attention as a multilevel selection process. *Cogn. Affect. Behav. Neurosci.* 4 483–500. 10.3758/CABN.4.4.483 15849892

[B39] Kastner S. Ungerleider L. G. (2000). Mechanisms of visual attention in the human cortex. *Annu. Rev. Neurosci.* 23 315–341. 10.1146/annurev.neuro.23.1.315 10845067

[B40] Keizer A. W. Hommel B. Lamme V. A. (2015). Consciousness is not necessary for visual feature binding. *Psychon. Bull. Rev.* 22 453–460. 10.3758/s13423-014-0706-2 25134470

[B41] Koene A. R. Zhaoping L. (2007). Feature-specific interactions in salience from combined feature contrasts: evidence for a bottom–up saliency map in V1. *J. Vis.* 7 6.1–14. 17685802 10.1167/7.7.6

[B42] Komban S. J. Kremkow J. Jin J. Wang Y. Lashgari R. Li X. (2014). Neuronal and perceptual differences in the temporal processing of darks and lights. *Neuron* 82 224–234. 10.1016/j.neuron.2014.02.020 24698277 PMC3980847

[B43] Krüger A. Tünnermann J. Scharlau I. (2016). Fast and conspicuous? Quantifying salience with the theory of visual attention. *Adv. Cogn. Psychol.* 12 20–38. 10.5709/acp-0184-1 27168868 PMC4862317

[B44] Krummenacher J. Grubert A. Töllner T. Müller H. J. (2014). Salience-based integration of redundant signals in visual pop-out search: evidence from behavioral and electrophysiological measures. *J. Vis.* 14 26–26. 10.1167/14.3.26 24648196

[B45] Lashgari R. Li X. Chen Y. Kremkow J. Bereshpolova Y. Swadlow H. A. (2012). Response properties of local field potentials and neighboring single neurons in awake primary visual cortex. *J. Neurosci.* 32 11396–11413. 10.1523/JNEUROSCI.0429-12.2012 22895722 PMC3436073

[B46] Lee J. Ahn J. H. (2012). Attention to banner ads and their effectiveness: an eye-tracking approach. *Int. J. Electron. Commer.* 17 119–137. 10.2753/JEC1086-4415170105

[B47] Li X. Chen Y. Lashgari R. Bereshpolova Y. Swadlow H. A. Lee B. B. (2015). Mixing of chromatic and luminance retinal signals in primate area V1. *Cereb. Cortex* 25 1920–1937. 10.1093/cercor/bhu002 24464943 PMC4459291

[B48] Li Z. (2002). A saliency map in primary visual cortex. *Trends Cogn. Sci.* 6 9–16. 10.1016/S1364-6613(00)01817-9 11849610

[B49] Liu H. Agam Y. Madsen J. R. Kreiman G. (2009). Timing, timing, timing: fast decoding of object information from intracranial field potentials in human visual cortex. *Neuron* 62 281–290. 10.1016/j.neuron.2009.02.025 19409272 PMC2921507

[B50] Mannion D. J. Donkin C. Whitford T. J. (2017). No apparent influence of psychometrically-defined schizotypy on orientation-dependent contextual modulation of visual contrast detection. *PeerJ* 5:e2921. 10.7717/peerj.2921 28149692 PMC5267566

[B51] Maunsell J. H. (1995). The brain’s visual world: representation of visual targets in cerebral cortex. *Science* 270 764–769. 10.1126/science.270.5237.764 7481763

[B52] May K. A. Zhaoping L. (2009). Effects of surrounding frame on visual search for vertical or tilted bars. *J. Vis.* 9:20. 10.1167/9.13.20 20055553

[B53] Motter B. C. (1993). Focal attention produces spatially selective processing in visual cortical areas V1, V2, and V4 in the presence of competing stimuli. *J. Neurophysiol.* 70 909–919. 10.1152/jn.1993.70.3.909 8229178

[B54] Mullen K. T. (2002). Differential distributions of red–green and blue–yellow cone opponency across the visual field. *Vis. Neurosci.* 19 109–118. 10.1017/S0952523802191103 12180855

[B55] Neokleous K. Shimi A. Avraamides M. N. (2016). Modeling the effects of perceptual load: saliency, competitive interactions, and top-down biases. *Front. Psychol.* 7:1. 10.3389/fpsyg.2016.00001 26858668 PMC4726798

[B56] Nordfang M. Wolfe J. M. (2014). Guided search for triple conjunctions. *Atten. Percept. Psychophys.* 76 1535–1559. 10.3758/s13414-014-0715-2 25005070 PMC5565881

[B57] Nothdurft H. C. (1992). Feature analysis and the role of similarity in preattentive vision. *Atten. Percept. Psychophys.* 52 355–375. 10.3758/BF03206697 1437470

[B58] Nothdurft H. C. (1993). Saliency effects across dimensions in visual search. *Vis. Res.* 33 839–844. 10.1016/0042-6989(93)90202-8 8351854

[B59] Nothdurft H. C. (2000a). Salience from feature contrast: additivity across dimensions. *Vis. Res.* 40 1183–1201. 10788635 10.1016/s0042-6989(00)00031-6

[B60] Nothdurft H. C. (2000b). Salience from feature contrast: temporal properties of saliency mechanisms. *Visi. Res.* 40 2421–2435. 10915883 10.1016/s0042-6989(00)00112-7

[B61] Nothdurft H. C. (2000c). Salience from feature contrast: Variations with texture density. *Vis. Res.* 40 3181–3200. 11008137 10.1016/s0042-6989(00)00168-1

[B62] Nothdurft H. C. (2002). Attention shifts to salient targets. *Vis. Res.* 42 1287–1306. 10.1016/S0042-6989(02)00016-0 12044759

[B63] Pashler H. (1998). *The Psychology of Attention.* Cambridge, MA: MIT press.

[B64] Pashler H. Dobkins K. Huang L. (2004). Is contrast just another feature for visual selective attention? *Vis. Res.* 44 1403–1410. 15066399 10.1016/j.visres.2003.11.025

[B65] Peck C. J. Jangraw D. C. Suzuki M. Efem R. Gottlieb J. (2009). Reward modulates attention independently of action value in posterior parietal cortex. *J. Neurosci.* 29 11182–11191. 10.1523/JNEUROSCI.1929-09.2009 19741125 PMC2778240

[B66] Pedale T. Santangelo V. (2015). Perceptual salience affects the contents of working memory during free-recollection of objects from natural scenes. *Front. Hum. Neurosci.* 9:60. 10.3389/fnhum.2015.00060 25741266 PMC4330792

[B67] Phillips S. Takeda Y. Kumada T. (2006). An inter-item similarity model unifying feature and conjunction search. *Vis. Res.* 46 3867–3880. 10.1016/j.visres.2006.06.016 16920177

[B68] Pieters R. Wedel M. (2004). Attention capture and transfer in advertising: brand, pictorial, and text-size effects. *J. Mark.* 68 36–50. 10.1509/jmkg.68.2.36.27794

[B69] Pinto Y. van der Leij A. R. Sligte I. G. Lamme V. A. Scholte H. S. (2013). Bottom-up and top-down attention are independent. *J. Vis.* 13:16. 10.1167/13.3.16 23863334

[B70] Poirier F. J. Gosselin F. Arguin M. (2008). Perceptive fields of saliency. *J. Vis.* 8:14. 10.1167/8.15.14 19146298

[B71] Pollmann S. Zinke W. Baumgartner F. Geringswald F. Hanke M. (2014). The right temporo-parietal junction contributes to visual feature binding. *Neuroimage* 101 289–297. 10.1016/j.neuroimage.2014.07.021 25038438

[B72] Pramod R. T. Arun S. P. (2014). Features in visual search combine linearly. *J. Vis.* 14:6. 10.1167/14.4.6 24715328 PMC3980647

[B73] Reynolds J. H. Chelazzi L. (2004). Attentional modulation of visual processing. *Annu. Rev. Neurosci.* 27 611–647. 10.1146/annurev.neuro.26.041002.131039 15217345

[B74] Rosbergen E. Pieters R. Wedel M. (1997). Visual attention to advertising: a segment-level analysis. *J. Consum. Res.* 24 305–314. 10.1086/209512

[B75] Rosenholtz R. (2001). Search asymmetries? What search asymmetries? *Percept. Psychophys.* 63 476–489. 10.3758/BF03194414 11414135

[B76] Santangelo V. (2015). Forced to remember: when memory is biased by salient information. *Behav. Brain Res.* 283 1–10. 10.1016/j.bbr.2015.01.013 25595422

[B77] Santangelo V. Di Francesco S. A. Mastroberardino S. Macaluso E. (2015). Parietal cortex integrates contextual and saliency signals during the encoding of natural scenes in working memory. *Hum. Brain Mapp.* 36 5003–5017. 10.1002/hbm.22984 26333392 PMC6869543

[B78] Santangelo V. Macaluso E. (2013). Visual salience improves spatial working memory via enhanced parieto-temporal functional connectivity. *J. Neurosci.* 33 4110–4117. 10.1523/JNEUROSCI.4138-12.2013 23447619 PMC3695392

[B79] Scialfa C. T. Esau S. P. Joffe K. M. (1998). Age, target-distractor similarity, and visual search. *Exp. Aging Res.* 24 337–358. 10.1080/036107398244184 9783154

[B80] Serences J. T. Yantis S. (2006). Selective visual attention and perceptual coherence. *Trends Cogn. Sci.* 10 38–45. 10.1016/j.tics.2005.11.008 16318922

[B81] Shiffrin R. M. Gardner G. T. (1972). Visual processing capacity and attentional control. *J. Exp. Psychol.* 93 72–82. 10.1037/h0032453 5013342

[B82] Sireteanu R. Encke I. Bachert I. (2005). Saliency and context play a role in infants’ texture segmentation. *Vis. Res.* 45 2161–2176. 10.1016/j.visres.2005.02.003 15845247

[B83] Sporns O. Tononi G. Edelman G. M. (1991). Modeling perceptual grouping and figure-ground segregation by means of active reentrant connections. *Proc. Natl. Acad. Sci. U.S.A.* 88 129–133. 10.1073/pnas.88.1.129 1986358 PMC50763

[B84] Stuit S. M. Verstraten F. A. Paffen C. L. (2010). Saliency in a suppressed image affects the spatial origin of perceptual alternations during binocular rivalry. *Vis. Res.* 50 1913–1921. 10.1016/j.visres.2010.06.014 20600231

[B85] Szczepanski S. M. Konen C. S. Kastner S. (2010). Mechanisms of spatial attention control in frontal and parietal cortex. *J. Neurosci.* 30 148–160. 10.1523/JNEUROSCI.3862-09.2010 20053897 PMC2809378

[B86] Tajima S. Komine K. (2015). Saliency-based color accessibility. *IEEE Trans. Image Process.* 24 1115–1126. 10.1109/TIP.2015.2393056 25608304

[B87] Töllner T. Zehetleitner M. Gramann K. Müller H. J. (2011). Stimulus saliency modulates pre-attentive processing speed in human visual cortex. *PLoS One* 6:e16276. 10.1371/journal.pone.0016276 21283699 PMC3025013

[B88] Treisman A. M. Gelade G. (1980). A feature-integration theory of attention. *Cognit. Psychol.* 12 97–136. 10.1016/0010-0285(80)90005-5 7351125

[B89] Treue S. (2003). Visual attention: the where, what, how and why of saliency. *Curr. Opin. Neurobiol.* 13 428–432. 10.1016/S0959-4388(03)00105-3 12965289

[B90] Tsotsos J. K. (1997). Limited capacity of any realizable perceptual system is a sufficient reason for attentive behavior. *Conscious. Cogn.* 6 429–436. 10.1006/ccog.1997.0302 9245465

[B91] Turatto M. Galfano G. (2000). Color, form and luminance capture attention in visual search. *Vis. Res.* 40 1639–1643. 10.1016/S0042-6989(00)00061-4 10814751

[B92] Turatto M. Galfano G. (2001). Attentional capture by color without any relevant attentional set. *Percept. Psychophys.* 63 286–297. 10.3758/BF03194469 11281103

[B93] Vijfvinkel A. (2014). *The Effect of Basic Features on Visual Saliency in (Branded) Objects.* Master thesis, Tilburg University, Tilburg.

[B94] Wang Y. Jin J. Kremkow J. Lashgari R. Komban S. J. Alonso J. M. (2015). Columnar organization of spatial phase in visual cortex. *Nat. Neurosci.* 18 97–103. 10.1038/nn.3878 25420070 PMC4281281

[B95] Ward R. Arend I. (2012). Feature binding across different visual dimensions. *Atten. Percept. Psychophys.* 74 1406–1415. 10.3758/s13414-012-0331-y 22688495

[B96] White B. J. Kan J. Y. Levy R. Itti L. Munoz D. P. (2017). Superior colliculus encodes visual saliency before the primary visual cortex. *Proc. Natl. Acad. Sci. U.S.A.* 114 9451–9456. 10.1073/pnas.1701003114 28808026 PMC5584409

[B97] Wolf J. M. Horowitz T. S. (2004). What attributes guide the deployment of visual attention and how do they do it? *Nat. Rev. Neurosci.* 5 495–501. 10.1038/nrn1411 15152199

[B98] Wolfe J. M. (1998). Visual search. *Attention* 1 13–73.

[B99] Wolfe J. M. Cave K. R. (1999). The psychophysical evidence for a binding problem in human vision. *Neuron* 24 11–17. 10.1016/S0896-6273(00)80818-1 10677023

[B100] Wool L. E. Komban S. J. Kremkow J. Jansen M. Li X. Alonso J. M. (2015). Salience of unique hues and implications for color theory. *J. Vis.* 15:10. 10.1167/15.2.10 25761328 PMC4319534

[B101] Wykowska A. Schubö A. (2010). On the temporal relation of top–down and bottom–up mechanisms during guidance of attention. *J. Cogn. Neurosci.* 22 640–654. 10.1162/jocn.2009.21222 19309292

[B102] Zehetleitner M. Hegenloh M. Müller H. J. (2011). Visually guided pointing movements are driven by the salience map. *J. Vis.* 11:24. 10.1167/11.1.24 21282341

[B103] Zehetleitner M. Koch A. I. Goschy H. Müller H. J. (2013). Salience-based selection: Attentional capture by distractors less salient than the target. *PLoS One* 8:e52595. 10.1371/journal.pone.0052595 23382820 PMC3557287

[B104] Zhang X. Qiu J. Zhang Y. Han S. Fang F. (2014). Misbinding of color and motion in human visual cortex. *Curr. Biol.* 24 1354–1360. 10.1016/j.cub.2014.04.045 24856212

[B105] Zhang X. Zhaoping L. Zhou T. Fang F. (2012). Neural activities in V1 create a bottom-up saliency map. *Neuron* 73 183–192. 10.1016/j.neuron.2011.10.035 22243756

[B106] Zhang Y. Zhang X. Wang Y. Fang F. (2016). Misbinding of color and motion in human early visual cortex: Evidence from event-related potentials. *Vis. Res.* 122 51–59. 10.1016/j.visres.2015.12.010 27038562

[B107] Zhaoping L. (2008). Attention capture by eye of origin singletons even without awareness—A hallmark of a bottom-up saliency map in the primary visual cortex. *J. Vis.* 8:1. 10.1167/8.5.1 18842072

[B108] Zhaoping L. May K. A. (2007). Psychophysical tests of the hypothesis of a bottom-up saliency map in primary visual cortex. *PLoS Comput. Biol.* 3:e62. 10.1371/journal.pcbi.0030062 17411335 PMC1847698

[B109] Zou B. Utochkin I. S. Liu Y. Wolfe J. M. (2017). Binocularity and visual search—Revisited. *Atten. Percept. Psychophys.* 79 473–483. 10.3758/s13414-016-1247-8 27900725

